# Desert date seed extract-loaded chitosan nanoparticles ameliorate hyperglycemia and insulin deficiency through the reduction in oxidative stress and inflammation

**DOI:** 10.1038/s41598-024-56352-3

**Published:** 2024-03-09

**Authors:** Alyaa Farid, Alaa Ahmed, Olaya Alaa, Gehan Safwat

**Affiliations:** 1 Biotechnology Department, Faculty of Science, Cairo University, Giza, Egypt; 2 Faculty of Biotechnology, October University for Modern Sciences and Arts (MSA), Giza, Egypt

**Keywords:** *Balanites aegyptiaca*, Chitosan, Diabetes mellitus, Beta cells, Cytokines, Immunochemistry, Nanobiotechnology, Nanoparticles, Drug delivery, Pharmacology

## Abstract

Plants represents a huge source of bioactive materials that have been used since the old times in the treatment of many diseases. *Balanites aegyptiaca*, known as desert date, has been used in treatment of fever, diabetes and bacterial infection. Desert dates contains a hard seed that resembles 50–60% of the fruit. The seed extract contains many fatty acids, amino acids and other bioactive materials that gives the extract its antioxidant and anti-inflammatory properties. The study aimed to use *Balanites* seed extract-loaded chitosan nanoparticles (SeEx-C NPs) for the treatment of streptozotocin (STZ)-induced diabetes in male Sprague Dawley rats. Animals were divided into two main divisions (healthy and diabetic rats). Each division contained seven groups (5 rats/group): control untreated group I, SeEx treated group II and group III (10 and 20 mg/kg b.w., respectively), C NPs treated group IV and group V (10 and 20 mg/kg b.w., respectively) and SeEx-C NPs treated group VI and group VII (10 and 20 mg/kg b.w., respectively). The therapeutical effects of SeEx-C NPs were evaluated through biochemical and immunological assessments in rats’ pancreases. The results showed that SeEx-C NPs (10 and 20 mg/kg b.w.) reduced the oxidative stress and inflammation in rats’ pancreases allowing the islets neogenesis. The loading of SeEx on C NPs allowed the delivery of fatty acids (oleic, lauric and myristic acid), amino acids (lysine, leucine, phenylalanine and valine) and minerals to pancreatic beta-cells in a sustainable manner. SeEx-C NPs administration successfully increased insulin secretion, allowed pancreatic islets neogenesis and reduced oxidative stress and inflammation.

## Introduction

Diabetes mellitus is a chronic disease affecting a huge part of each community. Its hallmarks are hyperglycemia that is accompanied with insulin resistance and/or production^1^. Nearly 382 million persons were affected in 2013, and by the year 2035, that number is projected to rise to 592 million. Since insulin is a treatment option for type 1 and late stage type 2 diabetic patients, its drawbacks have been reported in several researches^2^. According to Ogbera et al*.*^3^, diabetic patients began to use alternative and complementary therapies in an attempt to control elevated blood glucose level and enhance insulin secretion. According to Murthy et al*.*^4^, a number of medicinal plants have shown hypoglycemic effects. These effects appear to be achieved by the enhanced insulin production that results from the stimulation of pancreatic cells, inhibiting the absorption of glucose, or by increasing the insulin sensitivity. *Balanites aegyptiaca* (L.) Delile, belongs to Family: Zygophyllaceae, grows in the Arabian Peninsula, North East Africa (Egypt) and African Savannah region. It may be found in a number of habitats and tolerates a wide range of climatic moisture levels (from desert to subhumid areas), as well as, a wide range of soil types (sandy to heavy clay soil). According to Chothani and Vaghasiya^5^, it can tolerate flooding, cattle activities, and wildfires to a great extent.

Kamel et al*.*^6^ showed the hypoglycemic impact of an oral administration of an aqueous fruit extract in streptozotocin (STZ)-induced diabetic animals. According to Al-Malki et al*.*^7^, ethyl acetate extract containing beta-sitosterol regulated the oxidative damage brought on by STZ injection. In response to fruit extract administration to diabetic rats, Hassanin et al*.*^8^ reported a reduction in levels of glucose, haemoglobin A1c, lipid profile, and malondialdehyde (MDA). This resulted in a rise in insulin levels and a decrease in glutathione (GSH) levels, as well as, superoxide dismutase (SOD) and catalase (CAT) activity. They, also, noticed an amplification in the level of insulin receptor substrate 1 and a decrease in the levels of apoptosis signal-regulating kinase 1 (ASK1), protein 53 and c-Jun N-terminal kinase (JNK) in the animal’s pancreatic tissue.

A range of phytochemicals can be obtained from *Balanites* (fruits, leaves, seeds, stem bark, and roots) including phenolic and alkaloid compounds^4^. *Balanites* has a very hard fibrous seed (1.5–3 cm) with a light brown colour and accounts for 50–60% of the fruit. According to Mohamed et al*.*^9^, seeds have a substantial amount of protein, carbs and a 49% oil content. The main fatty acids in seed oil, which is utilised for dietary purposes, are linoleic, oleic, palmitic, and stearic acids. In addition to being used as a laxative, seed oil is used to treat epileptic seizures, haemorrhoids, stomach problems, jaundice, yellow fever, and syphilis. According to Molla et al*.*^10^, seed extract showed a molluscicidal activity against adult *Biomphalaria pfeifferi* and *Lymnaea natalensis*. Rat diets with 100 mg/kg b.w. of seed oil reduced lipid peroxidation and nitrogen oxide. Additionally, there was a down regulation in the messenger RNA (mRNA) expression of interleukin (IL)-6 and tumour necrosis factor (TNF)-α indicating an anti-inflammatory effect. According to Beit-Yannai et al*.*^11^, the saponin balanitin-7 (extracted from the seeds) exhibited an anti-cancer action.

Chitosan nanoparticles is biologically compatible with a high absorptive properties and rapid solubility; in addition to its ease of synthesis which make it a suitable carrier for a lot of drugs in medical applications^12^. Savin et al.^13^ reported that drug and bioactive molecule penetration, permeability, and sustained release can all be enhanced by chitosan-based hydrogels. Chitosan-based systems have demonstrated significant potential as alternative medications to treat many diseases since they are pH-sensitive, bioavailable, and non-toxic^14,15^.

The study aimed to use *Balanites* seed extract (SeEx) for the treatment of STZ-induced diabetes in male Sprague Dawley rats. The chemical composition of the seed extract inspired the idea of loading these bioactive constituents on chitosan nanoparticles (C NPs) to enhance their delivery and sustainable release. Chitosan (C) was chosen due to its recorded medicinal benefits including safety, biodegradable, antioxidant and anti-inflammatory properties. STZ-induced diabetic model is characterized by excessive oxidative stress and inflammation in pancreas that leads to islets beta cell damage and decreased insulin production. The therapeutical effects of SeEx-loaded C NPs (SeEx-C NPs) were evaluated through biochemical [fasting blood glucose (FBG), insulin, fructosamine (FTA), C-peptide and antioxidant enzymes levels] and immunological (pro-inflammatory cytokines) assessments in rats’ pancreases. The possibility of pancreatic islets’s beta cells neogenesis was evaluated through histological examination of pancreases from all experimental groups.

## Materials and methods

### Preparation of seed extract

Fresh fruits of *Balanites* were collected from trees in the Faculty of Agriculture, Cairo University. The tree and fruits were identified by Professor Gehan Safwat (Professor of biotechnology). The voucher specimens were kept in the herbarium of Botany Department, Faculty of Science, Cairo University. All required approvals were obtained for the study, which complied with all relevant regulations. The seed was collected from the fruits and washed with distilled water. The seeds were dried in the oven (40 °C) for one hour, crushed and grinded into fine powder by the aid of an electric grinder (Panasonic, Japan). Seed powder was mixed with ethanol (80%) and stirred for two hours; then left for three days at room temperature. The extract was filtered followed by centrifugation at 1500 rpm for five minutes^16^. The supernatant was concentrated by a rotary evaporator (R-200, MERCK) to obtain crude SeEx.

### Characterization of SeEx and SeEx-C NPs

The chemical composition of seed was performed by the official methods of the association of official analytical chemist^17^ at the microanalytical center of Cairo University. Thermo Scientific's Trace Gas chromatography1310–ISQ mass spectrometer (GC–MS) were used to identify the fatty acids components of SeEx and SeEx-C NPs. Inductively Coupled Plasma-mass spectrometry (ICP-MS) analysis was used to identify the different minerals in SeEx. The amino acids composition in SeEx and SeEx-C NPs was determined by a Beckman 120-C amino acid analyzer.

### Preparation of SeEx-C NPs

The C solution was prepared by dissolving C in an aqueous solution of acetic acid (1%, v/v) to a final concentration of 2% (w/v). The C solution received an addition of sodium tripolyphosphate (TPP) solution with continuous mixing at room temperature for 1 h until C NPs is formed. SeEx (5 mg/ml) was mixed with TPP solution for the preparation of SeEx-C NPs^18^. The size and form of the produced NPs were determined using the transmission electron microscope (TEM) and scanning electron microscope (SEM), respectively.

### Characterization of SeEx-C NPs

The antioxidant activity of the prepared SeEx and SeEx-C NPs were evaluated from the free radical scavenging effect using 1,1-diphenyl-2-picryl hydrazyl (DPPH) according to Farid et al*.*^18^. Different concentrations of SeEx and SeEx-C NPs were mixed with DPPH solution and allowed to stand for 30 min at room temperature. With ascorbic acid used as a control, the absorbance (abs) was measured at 517 nm and the antioxidant capacity was calculated from the equation:

$$\mathrm{Scavenging\, capacity }\,(\mathrm{\%})=[\frac{\left(\mathrm{control\, abs}-\mathrm{sample\, abs}\right)}{\mathrm{control\, abs}}]\times 100$$

Prothrombin time (PT) and partial thromboplastin time (PTT) were measured to evaluate the anticoagulant activity of SeEx and SeEx-C NPs with the use of heparin as a control^18^. Membrane stabilizing assay, using rats’ red blood cells, was used to assess the anti-inflammatory capacity of SeEx and SeEx-C NPs. Different concentrations were mixed with distilled water (hypotonic solution) or saline (isotonic solution); with indomethacin used as a control. The formed mixtures were incubated at 37 °C for 60 min followed by a period of incubation (3 min). The released hemoglobin, in the supernatant, was measured at 540 nm.

$$\mathrm{Hemolysis\, inhibition }(\mathrm{\%})=[\frac{\left(\mathrm{sample\, abs\, in\, hypotonic\, solution}-\mathrm{sample\, abs\, in\, isotonic\, solution}\right)}{\left(\mathrm{control\, abs\, in\, hypotonic\, solution}-\mathrm{ sample\, abs\, in\, isotonic\, solution}\right)}]\times 100$$

The MTT (3-(4,5-dimethylthiazol-2-yl)-2–5-diphenyltetrazolium bromide) assay was used to determine the cytotoxicity of prepared SeEx and SeEx-NPs^18^. Different concentrations were mixed with RPMI culturing media and added to Caco-2 cells monolayers in plates and incubated for 24 h at 37 °C in 5% CO_2_. After incubation, MTT was added to the cultures and the plates were incubated for another 4 h; followed by the addition of DMSO to solubilize formazan. The absorbance was read at 650 nm.

### Experimental design

The study was performed according to the international guidelines for the care and use of laboratory animals and in compliance with the ARRIVE guidelines. The study was approved by the ethics committee of October University for Modern Sciences and Arts (MSA 0323). Male Sprague Dawley rats (10 weeks and 170–200 g) were purchased from the National Organization for Drug Control and Research (Egypt) and were divided into two main divisions (healthy and diabetic rats). Each division contained seven groups (5 rats/group) as the following: Group I: control untreated rats, Group II: SeEx (10 mg/kg b.w.) treated rats, Group III: SeEx (20 mg/kg b.w.) treated rats, Group IV: C NPs (10 mg/kg b.w.) treated rats, Group V: C NPs (20 mg/kg b.w.) treated rats, Group VI: SeEx-C NPs (10 mg/kg b.w.) treated rats and Group VII: SeEx-C NPs (20 mg/kg b.w.) treated rats.

Diabetes induction was performed by the intravenous injection of STZ (65 mg/kg b.w.) after 6 h of fasting (water was provided ad libitum through the fasting hours)^19^. Rats were provided with sucrose dissolved in drinking water (10%) on the first day after STZ injection. Fasting blood glucose (FBG) level was monitored for ten days, where, rats with FBG level more than 150 mg/dl were considered diabetic. SeEx, C NPs and SeEx-C NPs were orally administrated via intragastric tube daily to all experimental groups for 8 weeks. At the end of the study, rats were anesthetized with sodium pentobarbital (80 mg/kg b.w.). Blood samples were collected through cardiac puncture for the preparation of serum. Rats were dissected to extract the pancreases for the biochemical and histopathological examination. Pancreatic tissue homogenates were prepared by the homogenization of pancreatic tissue (1 g) with cold tris–HCl (4.5 ml). The homogenates were centrifuged and the protein content in the supernatant was measured by the Lowry’s method^20^. FTA level was measured by ELISA (MBS2601586, MyBioSource, USA) to evaluated the glycated serum protein that indicate the rats’ glucose level over the past 2–3 weeks. Serum insulin level was measured by ELISA (Invitrogen, ERINS) to evaluate the effect of treatment on rats’ glycemia. Moreover, serum C-peptide level was measured by CPT11-K01 ELISA kit (Eagle Biosciences, USA). Homeostatic Model Assessment for Insulin Resistance (HOMA-IR) and quantitative insulin sensitivity check index (QUICKI) were calculated for all experimental groups from the following formulas: HOMA-IR = [FBG (mmol/L)Xfasting insulin (mIU/L)]/22.5 and QUICKI = 1/[log(fasting insulin (mIU/L)) + log(FBG (mg/dl))], respectively^21^.

### Oxidative stress and immunological measurements

The oxidative stress in pancreatic tissue homogenates was assessed by measuring the antioxidant enzymes (CAT, SOD and GSH) by ELISA (MBS726781, MBS036924 and MBS265966; MyBioSource, USA). Lipid peroxidation in pancreas was evaluated through measuring MDA by ELISA (MBS268427, MyBioSource, USA). The effect of STZ administration and the anti-inflammatory effect of tested treatments in rat’s pancreas were demonstrated by measuring the pro-inflammatory cytokines (TNF-α, IL-1β, IL-6 and IL-4).

### Histopathological examination of rat’s pancreas

Pancreases were collected, from all experimental animals, fixed with buffered formalin (10%), dehydrated in an ascending grades of alcohol, cleared by xylene and embedded in paraffin blocks. By a microtome, pancreatic blocks were cut into 4 μm sections and stained with hematoxylin and eosin (H&E) stain^19^. For insulin immunohistochemical examination, pancreatic sections were deparrafinized, rehydrated and washed. To block the activity of endogenous peroxidases, H_2_O_2_ (3%) was added to pancreatic sections followed by bovine serum albumin (5%). Pancreatic sections were incubated, for 30 min, with primary antibody [anti-insulin monoclonal antibody (I2018, Sigma, USA)]; then, incubated, for 60 min, by a secondary antibody [HRP-rabbit anti-rat IgG (ab6734, abcam, USA)]. 3,3-diaminobenzidine (DAB) was added for the development of brown colour (positive result). Histopathological scoring of pancreatic damage (0–3) was as the following: Necrosis (0: absent, 1: focal necrosis, 2: partial necrosis, 3: total necrosis), inflammatory cells infiltration (0: absent, 1: mild, 2: moderate, 3: marked) and edema (0: absent, 1: mild, 2: moderate, 3: marked). The degree of pancreatic islets’ neogenesis and insulin secretion were evaluated by the morphometric analysis (0–2) as the following: islets size (0: atrophid, 1: small, 2: average), cellularity (0: acellular, 1: hypocellular, 2: average), beta cell damage (0: absent, 1: moderate, 2: marked) and insulin secretion (0: marked, 1: mild, 2: weak).

### Statistical analysis

Results were examined by one way analysis of variance (ANOVA) and compared with Tukey's homogeneity test. Results were expressed as mean ± standard deviation (SD), and considered significant when *P* value was less than 0.05.

### Ethics approval

All experimental procedures were carried out in accordance with the international guidelines for the care and use laboratory animals, and the study was conducted in accordance with the guide for the care and use of laboratory animals, Eighth edition (2011). The study has been approved by the ethics committee of October University for Modern Sciences and Arts.

## Results

### Chemical characterization of date seed

According to the proximate analysis, seed contained 6.4% protein, 16.5 crude fibers, 9.8% crude fats, 55.9% carbohydrates. 1.3% ash and a 7.6% moisture (Table 1). High levels of potassium and phosphorus (305.4 and 108.5 mg/100 g dry weight, respectively) were found in the date seed; followed by magnesium, calcium and sodium (61.2, 47.5 and 29.8 mg/100 g dry weight). Zinc, iron, manganese and copper were, also, found but with small amount (Table 1).

**Table 1:** Proximate analysis and minerals content in date seed.

Composition	*Balanites* seed
Proximate analysis (% (g/100 g dry weight)	
Moisture	7.6 ± 0.2
Protein	6.4 ± 1.2
Crude fibers	16.5 ± 2.1
Crude fats	9.8 ± 0.9
Ash	1.3 ± 0.1
Carbohydrates	55.9 ± 1.1
E nergy value (kcal/100 g dry wight)	330.5 ± 2.2
Minerals (mg/100 g dry weight)	
Magnesium	61.2 ± 0.7
Potassium	305.4 ± 3.1
Calcium	47.5 ± 0.8
Zinc	3.3 ± 0.2
Sodium	29.8 ± 1.1
Phosphorus	108.5 ± 0.8
Iron	6.9 ± 0.5
Manganese	2.7 ± 0.2
Copper	0.9 ± 0.1

Results were expressed as mean ± standard deviation.

The fatty acids analysis of SeEx and SeEx-C NPs showed the presence of high percent of oleic acid (37.82 and 37.79%, respectively) followed by lauric (19.72 and 19.69%, respectively) and myristic acids (13.82 and 13.81%, respectively). Also, considerable amounts of palmitic acid (9.98 and 9.92%, respectively), linoleic acid (7.77 and 7.73%, respectively), capric acid (3.11 and 3.1%, respectively), stearic acid (2.79 and 2.76%, respectively) and arachidic acid (1.27 and 1.25%, respectively) were detected. In addition to, negligible percents of pentadecanoic acid (0.04 and 0.04%, respectively), tridecanoic acid (0.03 and 0.03%, respectively), margaric acid (0.05 and 0.04%, respectively), eicosadienoic acid (0.09 and 0.08%, respectively) and eicosatetraenoic acid (0.04 and 0.03%, respectively) were found. Both of α- and γ-Linolenic acids were detected in date seeds (Table 2).

**Table 2:** Fatty acids composition in SeEx and SeEx-C NPs.

Fatty acid	% of total fatty acids	
	SeEx	SeEx-C NPs
Caproic acid (C6:0)	0.22 ± 0.1	0.21 ± 0.1
Caprylic acid (C8:0)	0.73 ± 0.1	0.72 ± 0.1
Capric acid (C10:0)	3.11 ± 0.2	3.1 ± 0.3
Lauric (C12:0)	19.72 ± 0.4	19.69 ± 0.2
Tridecanoic acid (C13:0)	0.03 ± 0.01	0.03 ± 0.01
Myristic acid (C14:0)	13.82 ± 0.1	13.81 ± 0.4
Pentadecanoic Acid (C15:0)	0.04 ± 0.01	0.04 ± 0.01
Palmitic acid (C16:0)	9.98 ± 0.4	9.92 ± 0.2
Margaric acid (C17:0)	0.05 ± 0.01	0.04 ± 0.01
Stearic acid (C18:0)	2.79 ± 0.3	2.76 ± 0.1
Arachidic acid (C20:0)	1.27 ± 0.7	1.25 ± 0.1
Behenic acid (C22:0)	0.49 ± 0.1	0.46 ± 0.1
Tricosylic acid (C23:0)	0.21 ± 0.1	0.19 ± 0.3
Lignoceric (C24:0)	0.11 ± 0.1	0.11 ± 0.2
Saturated fatty acids (SFA)	**52.57**	**52.33**
Myristoleic acid (C14:1)	0.71 ± 0.01	0.71 ± 0.02
Palmitoleic (C16:1)	0.23 ± 0.2	0.22 ± 0.1
Oleic acid (C18:1)	37.82 ± 0.5	37.79 ± 0.6
Gadoleic acid (C20:1)	0.22 ± 0.01	0.21 ± 0.02
Monounsaturated fatty acids (MUFA)	**38.98**	**38.93**
Linoleic acid (C18:2)	7.77 ± 0.1	7.73 ± 0.3
γ–Linolenic acid (C18:3)	0.24 ± 0.2	0.22 ± 0.1
α-Linolenic acid (C18:3)	0.31 ± 0.1	0.3 ± 0.2
Eicosadienoic acid (C20:2)	0.09 ± 0.01	0.08 ± 0.02
Eicosatetraenoic acid (C20:4)	0.04 ± 0.01	0.03 ± 0.01
**Polyunsaturated fatty acids (PUFA)**	**8.45**	**8.36**

Results were expressed as mean ± standard deviation.

Significant values are in [bold].

The concentration of non-essential amino acids in SeEx and SeEx-C NPs (63.31 and 61.85 g/100 g protein, respectively) was higher than that of essential amino acids (36.69 and 36.3 g/100 g protein, respectively). All nine essential amino acids were detected in SeEx and SeEx-C NPs (Table 3). SeEx and SeEx-C NPs contained high concentration of essential amino acids such as lysine (8.52 and 8.43 g/100 g protein, respectively), leucine (6.87 and 6.81 g/100 g protein, respectively), phenylalanine (4.86 and 4.84 g/100 g protein, respectively) and valine (4.69 and 4.64 g/100 g protein, respectively). Glutamic acid (19.46 and 18.39 g/100 g protein, respectively), arginine (12.35 and 12.31 g/100 g protein, respectively) and aspartic acid (9.56 and 9.49 g/100 g protein, respectively) constituted the highest concentration of non-essential amino acids; followed by alanine (5.47 and 5.43 g/100 g protein, respectively), serine (4.55 and 4.52 g/100 g protein, respectively), glycine (4.23 and 4.21 g/100 g protein, respectively) and proline (3.88 and 3.81 g/100 g protein, respectively).

**Table 3:** amino acids composition in SeEx and SeEx-C NPs.

Amino acids	g/100 g protein	
	SeEx	SeEx-C NPs
Histidine (His)	2.11 ± 1.1	2.1 ± 0.2
Isoleucine (Ile)	2.97 ± 0.9	2.92 ± 0.8
Leucine (Leu)	6.87 ± 0.8	6.81 ± 0.2
Lysine (lys)	8.52 ± 0.2	8.43 ± 0.1
Methionine (Met)	2.57 ± 0.3	2.53 ± 0.2
Phenylalanine (Phe)	4.86 ± 0.1	4.84 ± 0.5
Threonine (Thr)	2.99 ± 0.8	2.93 ± 0.4
Tryptophan (Trp)	1.11 ± 0.1	1.1 ± 0.1
Valine (Val)	4.69 ± 0.3	4.64 ± 0.2
Essential amino acids	36.69	36.3
Aspartic acid (Asp)	9.56 ± 0.7	9.49 ± 0.4
Serine (Ser)	4.55 ± 0.6	4.52 ± 0.2
Glutamic acid (Glu)	19.46 ± 0.8	18.39 ± 0.7
Proline (Pro)	3.88 ± 0.2	3.81 ± 0.8
Glycine (Gly)	4.23 ± 0.1	4.21 ± 0.1
Alanine (Ala)	5.47 ± 0.4	5.43 ± 0.3
Cysteine (Cys)	1.87 ± 0.2	1.79 ± 0.4
Tyrosine (Tyr)	1.94 ± 0.1	1.9 ± 0.2
Arginine (Arg)	12.35 ± 0.3	12.31 ± 0.1
Non-essential amino acids	63.31	61.85

Results were expressed as mean ± standard deviation.

### Characterization of SeEx-C NPs

**Figure 1:**
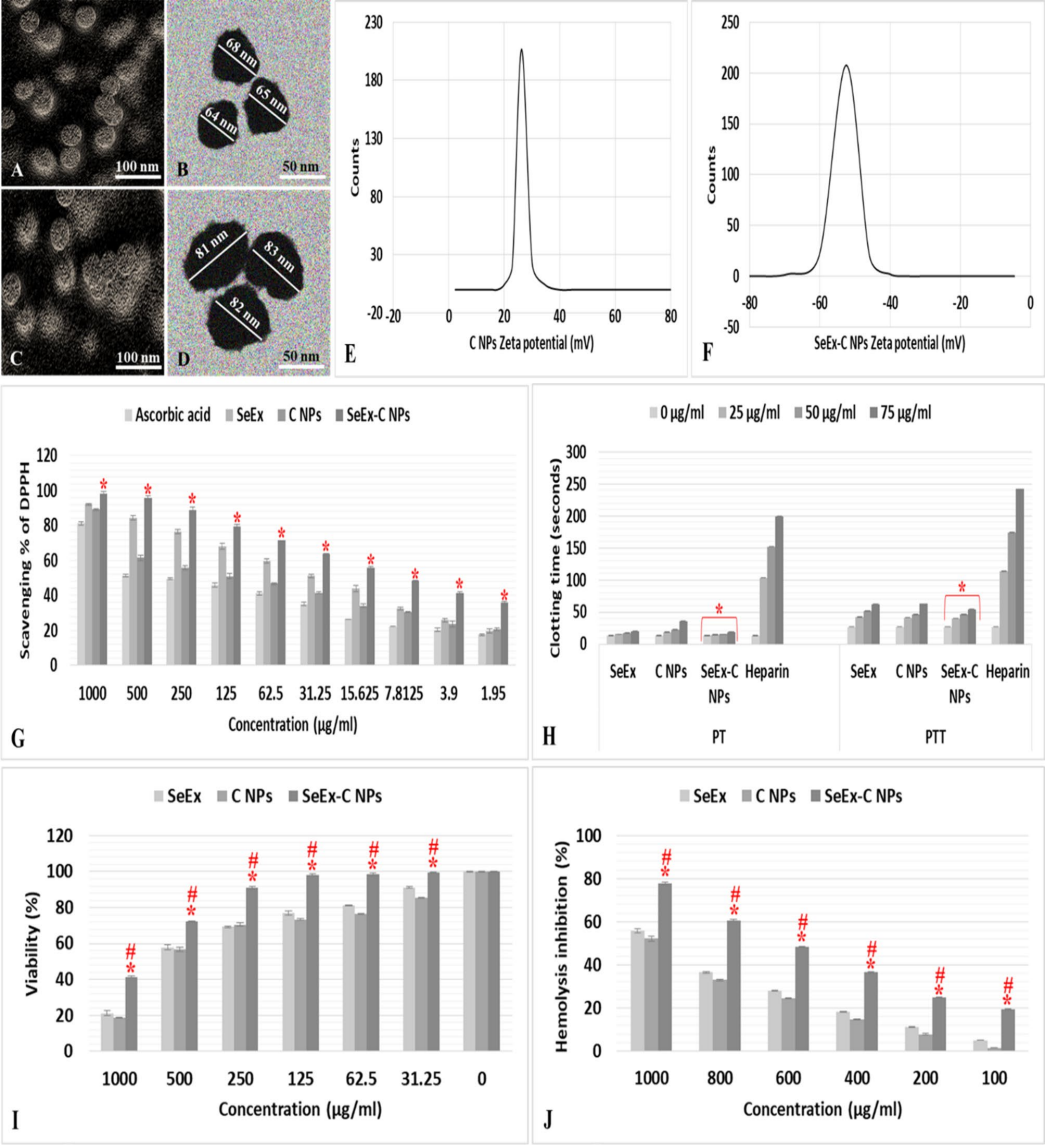
Shape and size of prepared C NPs and SeEx-C NPs by SEM (**A** and **C**) and TEM (**B** and **D**), Zeta potential of C NPs (**E**) and SeEx-C NPs (**F**), scavenging capacity % (**G**) (* represented significance (*p* < 0.05) in comparison to ascorbic acid), clotting time (**H**) (*represented significance (*p* < 0.05) in comparison to heparin), viability % (**I**) (*represented significance (*p* < 0.05) in comparison to SeEx and #represented significance (*p* < 0.05) in comparison to C NPs) and hemolysis inhibition % (**J**) (*represented significance (*p* < 0.05) in comparison to SeEx and #represented significance (*p* < 0.05) in comparison to C NPs).

SEM (Fig. 1A,C) and TEM (Fig. 1B,D) images of both C NPs and SeEx-C NPs showed a smooth spherical NPs with a size range of 63–68 and 81–83 nm, respectively. According to (Fig. 1E,F) the Zeta potential was 25 and − 55 mV for C NPs and SeEx-C NPs, respectively. SeEx-C NPs showed a high antioxidant effect than C NPs and SeEx in a dose dependent manner. This was evident from its high DPPH scavenging effect (Fig. 1G). SeEx-C NPs protected Caco-2 cells at high concentration (up to 250 μg/ml) and achieved high viability % (Fig. 1I); and showed high anti-inflammatory activity where it inhibited red blood cells’ hemolysis (Fig. 1J). The anti-coagulant effect of SeEx, C NPs and SeEx-C NPs increased with increasing the tested dose. PT and PTT of SeEx-C NPs were significantly less than those of heparin (Fig. 1H).

### Effect of SeEx, C NPs and SeEx-C NPs on body weight and hyperglycemia

**Figure 2:**
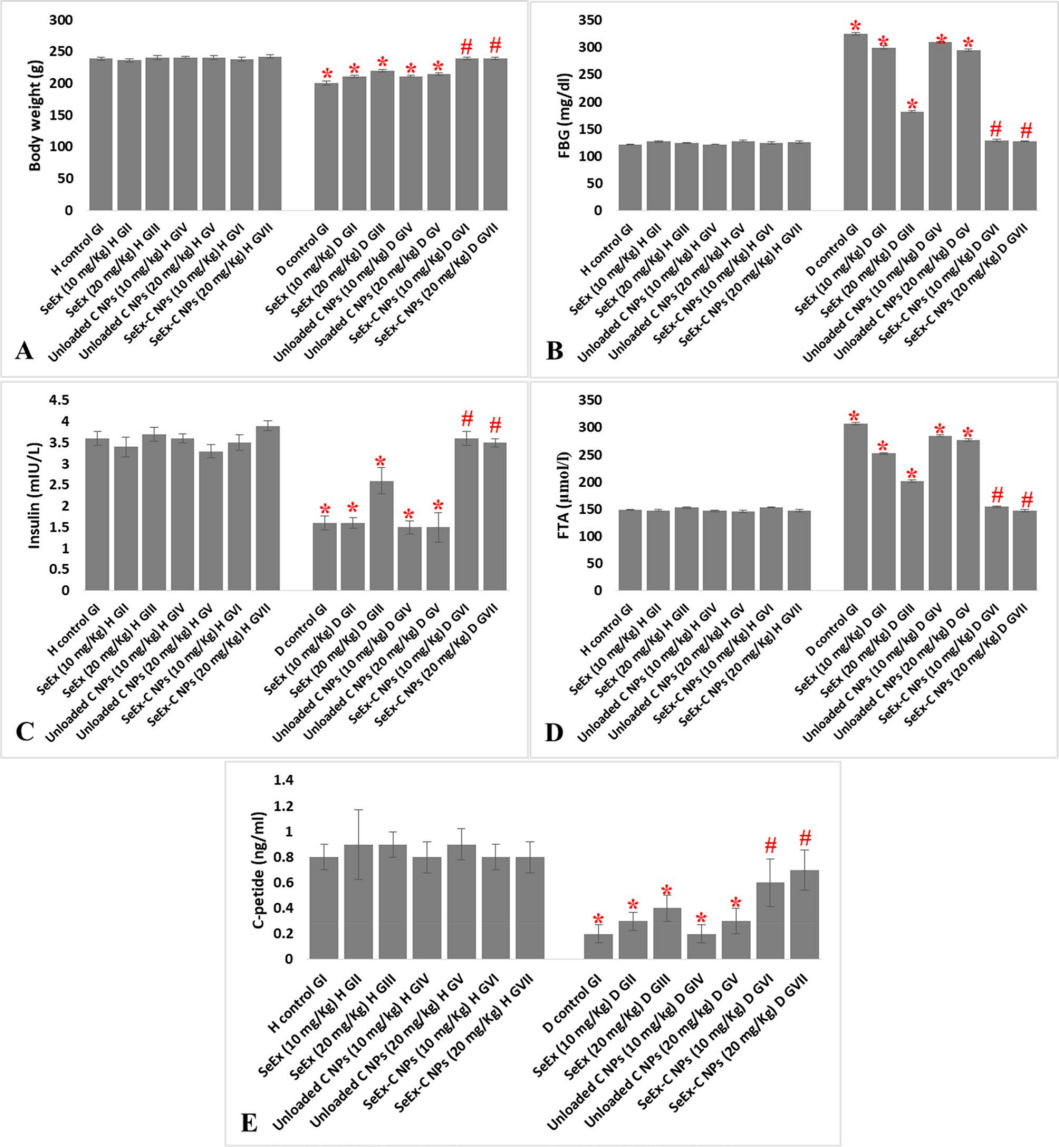
Effect of SeEx, C NPs and SeEx-C NPs on body weight (**A**), FBG (**B**), insulin (**C**), FTA (**D**) and C-peptide (**E**) levels. Results were expressed as mean ± SD, *represented significance when compared to healthy control (H control GI) and #represented significance when compared to untreated diabetic control (D control GI) (*p* < 0.05).

**Figure 3:**
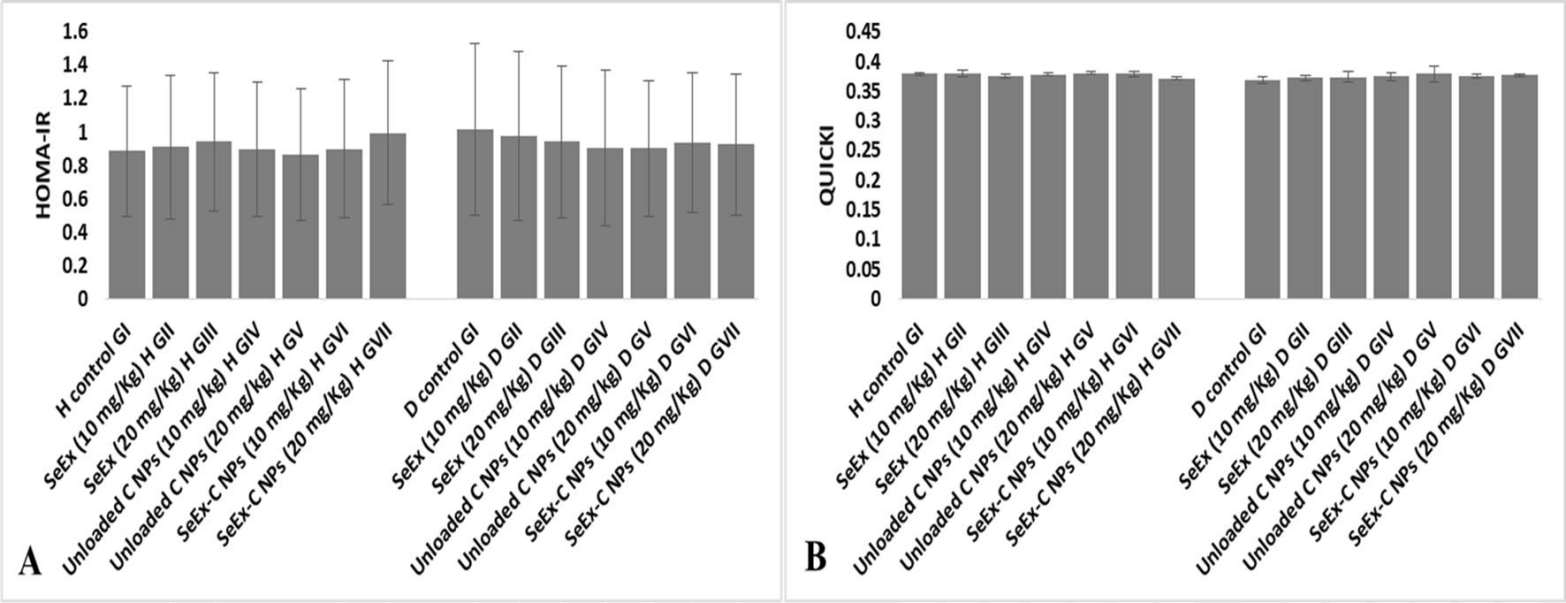
HOMA-IR and QUICKI in different experimental groups.

Diabetes induction significantly reduced animals’ body weight in untreated diabetic GI (200.6 g) when compared to healthy control GI (239.3 g). SeEx and C NPs (either 10 or 20 mg/kg b.w.) administration lead to an enhancement in animals’ body weight of treated diabetic GII (210.6), GIII (220.5 g), GIV (211.6 g) and GV (214.9 g), however, animals’ body weight remained significantly lower than that of healthy control GI (239.3 g). No significant difference was observed among healthy control GI and SeEx-C NPs treated groups (239.6 and 239.4 g for GVI and GVII). A significant reduction in insulin level and a significant elevation in FBG and FTA levels were noticed after diabetes induction in GI (1.6 ng/ml, 325.1 mg/ml and 307.3 μmol/l, respectively). Only, SeEx-C NPs (either 10 or 20 mg/kg b.w.) have succeeded in decreasing hyperglycemia and increasing insulin level in diabetic treated group. Where, no significant difference was observed in insulin, FBG and FTA levels among healthy control GI and SeEx-C NPs treated groups (Fig. 2). Serum C-peptide level was significantly reduced upon diabetes induction to reach (0.2 ng/ml) in untreated diabetic GI. SeEx administration raised C-peptide level in treated diabetic GII (0.3 ng/ml) and GIII (0.4 ng/ml), but, the levels remained significantly reduced when compared to healthy control GI (0.8 ng/ml). SeEx-C NPs treated diabetic GVI and GVII showed C-peptide levels (0.6 and 0.7, respectively) similar to those of healthy control groups. Figure 3 showed no significant difference in HOMA-IR or QUICKI among the different experimental groups.

### Effect of SeEx, C NPs and SeEx-C NPs on oxidative stress

**Figure 4:**
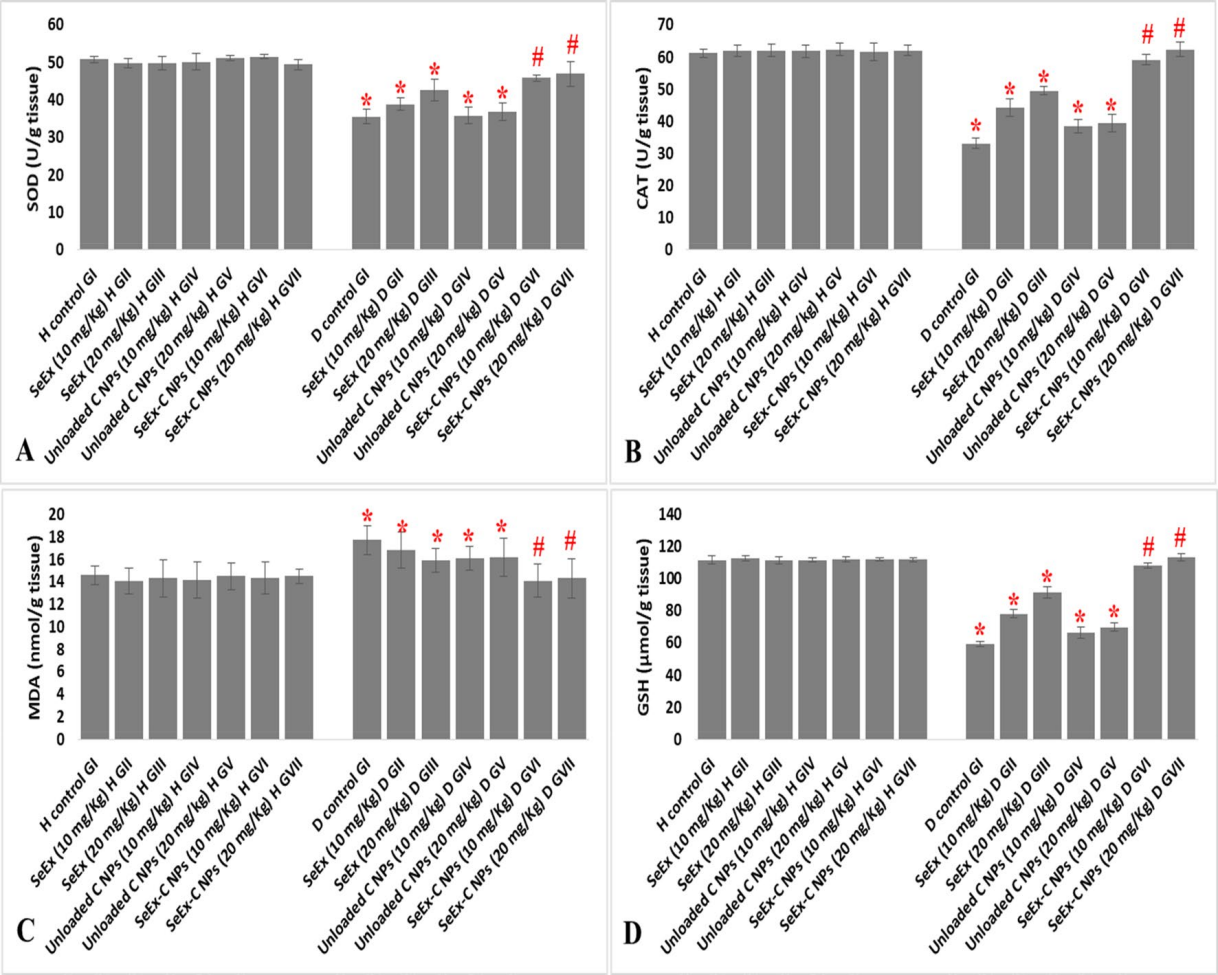
Effect of SeEx, C NPs and SeEx-C NPs on SOD (**A**), CAT (**B**), MDA (**C**) and GSH (**D**) levels. Results were expressed as mean ± SD, *represented significance when compared to healthy control (H control GI) and #represented significance when compared to untreated diabetic control (D control GI) (*p* < 0.05).

Diabetes induction, in untreated GI, significantly elevated MDA level (17.7 nmol/g tissue) and significantly reduced the antioxidant enzymes levels (35.6 U/g tissue, 33.1 U/g tissue and 59.1 μmol/g tissue for SOD, CAT and GSH, respectively) when compared to those of healthy control GI. SeEx administration (especially 20 mg/kg b.w.) showed better result than C NPs (either 10 or 20 mg/kg b.w.) administration. Only SeEx-C NPs administration significantly ameliorated the oxidative stress that results from STZ administration. Where, no significant difference was observed in MDA, SOD, CAT and GSH levels among healthy control GI and SeEx-C NPs treated groups Fig. 4.

### Effect of SeEx, C NPs and SeEx-C NPs on pro-inflammatory cytokines

**Figure 5:**
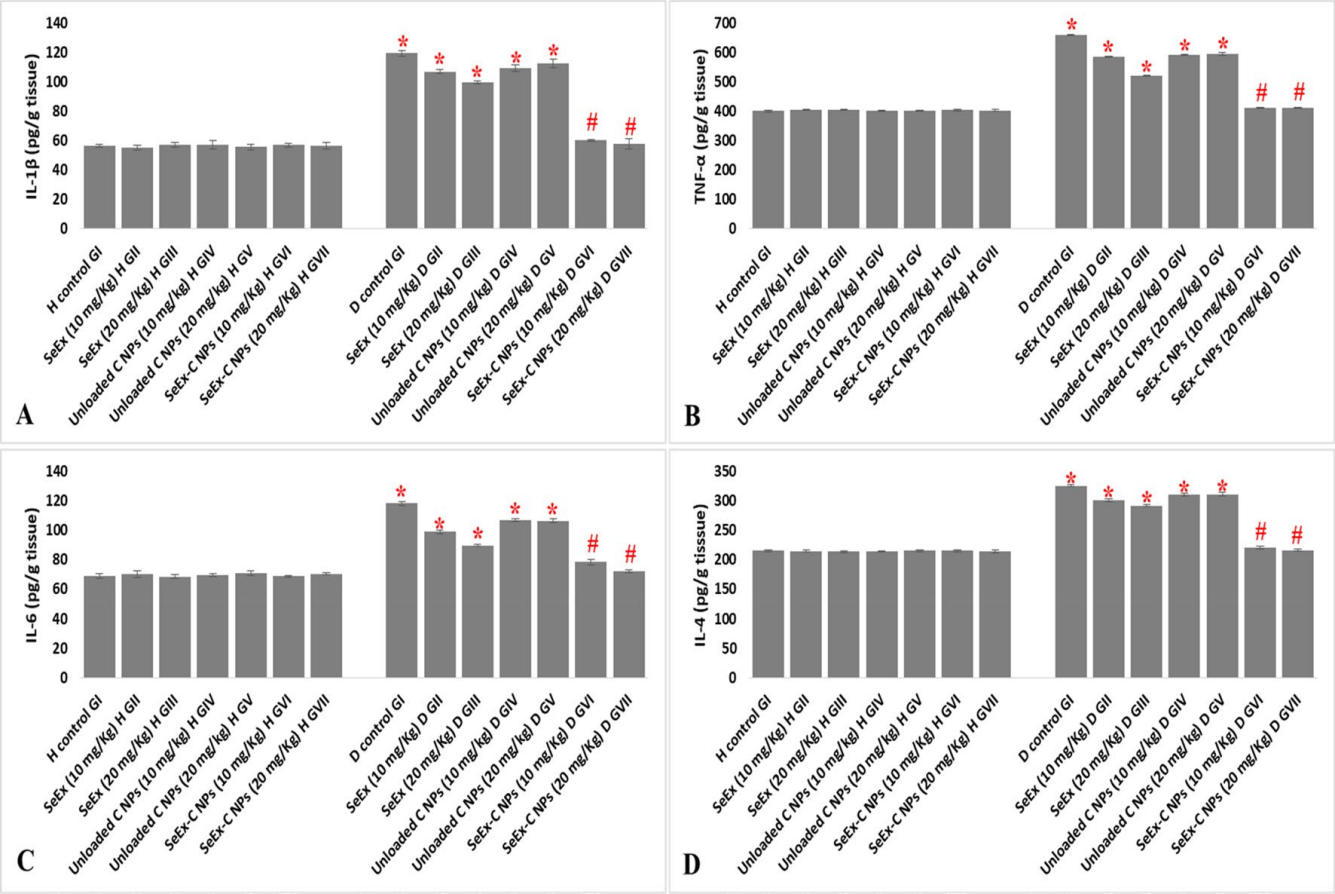
Effect of SeEx, C NPs and SeEx-C NPs on pro-inflammatory cytokines [IL-1β (**A**), TNF-α (**B**), IL-6 (**C**) and IL-4 (**D**)] levels. Results were expressed as mean ± SD, *represented significance when compared to healthy control (H control GI) and #represented significance when compared to untreated diabetic control (D control GI) (*p* < 0.05).

A significant elevation in pro-inflammatory cytokines (119.2, 659.9, 118.2 and 325.6 pg/g tissue for IL-1β, TNF-α, IL-6 and IL-4, respectively) was observed in diabetic control GI when compared to healthy control GI (56.6, 399.9, 69.2 and 215.2 pg/g tissue for IL-1β, TNF-α, IL-6 and IL-4, respectively). SeEx-C NPs showed significant anti-inflammatory effect, in vivo, in diabetic treated GVI and GVII, where, it significantly reduced the pro-inflammatory cytokines levels in those groups to be similar to those of healthy control GI (Fig. 5). On the other hand, SeEx and C NPs administration (either 10 or 20 mg/kg b.w.) were not effective in reducing the inflammation that resulted from diabetes induction by STZ (when compared to SeEx-C NPs).

### Histopathological examination

**Figure 6:**
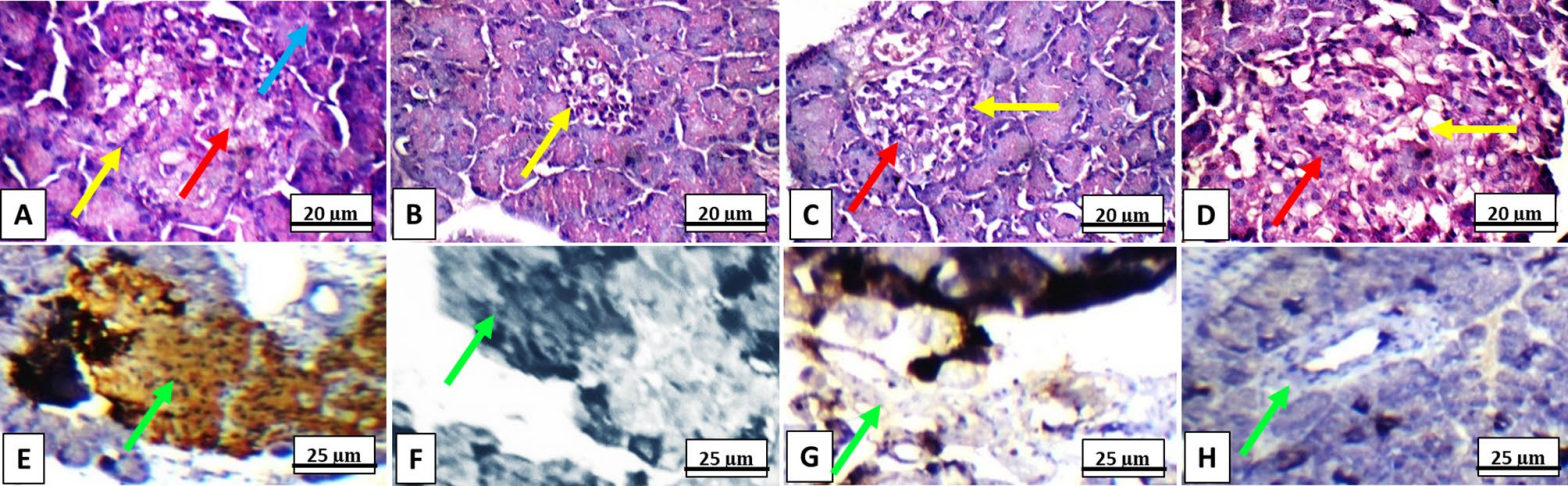
Rat’s pancreatic sections showing (**A**) average-sized normocellular islets of Langerhans (yellow arrow), with beta cells (red arrow), and average exocrine areas (blue arrow) in healthy control groups (I to VI) and diabetic groups treated with SeEx-C NPs (10 and 20 mg/kg b.w.) (H&E, × 400), (**B**) markedly damaged hypocellular islets of Langerhans (yellow arrow) in untreated diabetic control GI (H&E, × 400), (**C**) and (**D**) small-sized hypocellular islets (yellow arrow) with scattered apoptotic beta cells (red arrow) in diabetic groups treated with SeEx (10 and 20 mg/kg b.w.) and diabetic groups treated with C NPs (10 and 20 mg/kg b.w.) (H&E, × 400), (**E**) marked cytoplasmic reactivity (+ + +) for insulin (green arrow) in more than 90% of islet cells of healthy control groups (I–VI) and diabetic groups treated with SeEx-C NPs (10 and 20 mg/kg b.w.) (insulin immunostain, × 400), (**F**) negative cytoplasmic reactivity (−) for insulin (green arrow) of islet cells of untreated diabetic control GI (insulin immunostain, × 400), (**G**) and (**H**) weak cytoplasmic reactivity ( +) for insulin (green arrow) in less than 10% of islet cells of diabetic groups treated with SeEx (10 and 20 mg/kg b.w.) and diabetic groups treated with C NPs (10 and 20 mg/kg b.w.) (insulin immunostain, × 400).

**Figure 7:**
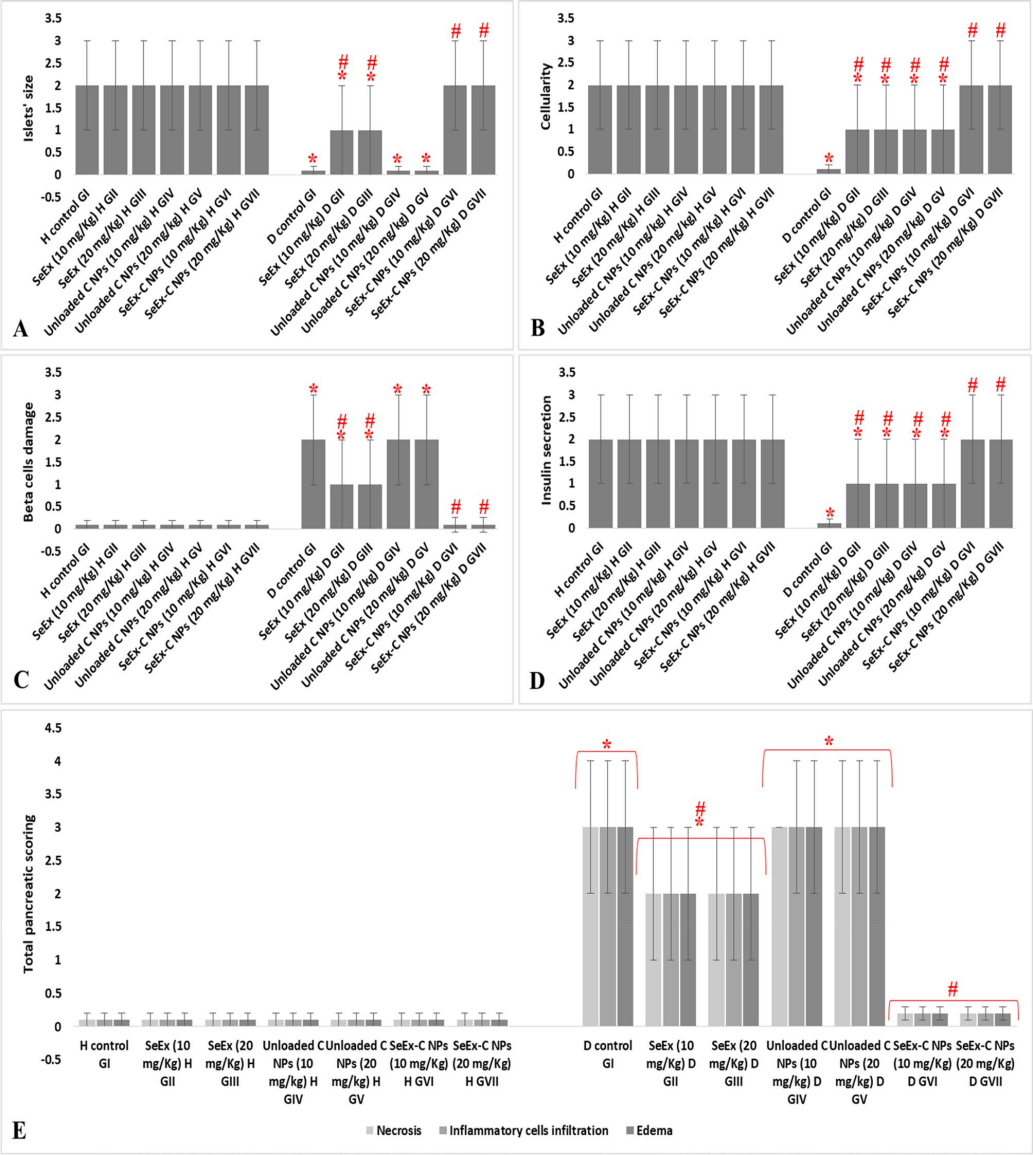
histopathological pancreatic scoring showing islets’ size (**A**), cellularity (**B**), beta cells damage (**C**) and insulin secretion (**D**); in addition to, morphometric analysis (**E**). Results were expressed as mean ± SD, *represented significance when compared to healthy control (H control GI) and #represented significance when compared to untreated diabetic control (D control GI) (*p* < 0.05).

Healthy control groups (GI-GVII) and SeEx-C NPs treated diabetic GVI and GVII showed average-sized normocellular islets of Langerhans with marked insulin immunostain (+ + +). On the other hand, untreated diabetic GI showed markedly damaged hypocellular islets of Langerhans with negative insulin immunostain (−). Diabetic groups treated with SeEx and C NPs (either 10 or 20 mg/kg b.w.) showed small-sized hypocellular islets with scattered apoptotic beta cells and average exocrine areas with weak insulin immunosatin (+) (Fig. 6). The pancreatic scoring and morphometric analysis indicated a reduction in islets’ size, cellularity and insulin secretion; in addition to, an elevation in beta cell damage, necrosis, inflammatory cells infiltration and edema in untreated diabetic GI. SeEx-C NPs administration ameliorated these histological alterations indicating islets cells neogenesis and increased insulin secretion Fig. 7.

## Discussion

Seeds are a by-product of the manufacture of fruit and are typically handled as litter, fed to animals, or simply thrown away^22^. But seeds, which make up 50–60% of the fresh weight of desert date fruit, are abundant in phytochemical ingredients^23^. Numerous research have been conducted on the pharmacological benefits of date seeds, including their anti-inflammatory, antioxidant, antidiabetic, antimicrobial, and antiviral characteristics^24^. Date seeds have a significant potential as a dietary therapeutic option for a number of chronic disorders because of their health benefits^25^. *Balanites*, a member of the Balantiaceae family, is commonly known as desert date according to Anwar and Aastha^26^. It is a wild tree that develops in the desert and savannah parts of Africa^27^. The seed is rich in fatty acids, amino acids, and minerals, that enables the use of the seed extract in many therapeutic approaches (antioxidant, anti-inflammation, antiviral, antimutagenic and antimicrobial).

In this study, *Balanites* seeds were extracted by ethanol (80%) to form crude extract. The seeds were chemically characterized to identify the contained different components that can be loaded on C NPs. The proximate study revealed that the seed has a 6.4% protein content, 16.5% crude fibres, 9.8% crude fats, and 55.9% carbohydrates. The seed included high concentrations of potassium (305.4 mg/100 g dry weight) and phosphorus (108.5 mg/100 g dry weight), which were followed by magnesium, calcium, and sodium (61.2, 47.5, and 29.8 mg/100 g dry weight). In minor amounts, zinc, iron, manganese, and copper were found as well. Alkhoori et al*.*^28^ reported that vitamins found in the seeds included vitamins A, B1 (thiamine), B2 (riboflavin), C, E, K, and folate. They added that seeds contains dietary fibres with functional properties, such as pectin, tannins, lignins, and hemicellulose. Seeds, contained several types of minerals, with K being the most prevalent^29^. Also, Na, Zn, P, F, Se, Ca, Cu, Co, Fe, Mg and B, were found in the seeds^30^. Consuming date seeds is advised for those with hypertension due to their low sodium level. Biglar et al*.*^31^ found that lauric acid, with a content of 18.78–31.61%, and oleic-lauric oil, with a content of 33.38–51.40%, were the two primary fatty acids in date seeds. According to reports, linoleic and palmitic acids are the first two primary fatty acids, with myristic acid coming in third^32^. Our results showed that oleic acid was found in significant concentrations in the fatty acids in SeEx and SeEx-C NPs, followed by lauric and myristic acids. Additionally, significant levels of arachidic acid, capric acid, stearic acid, linoleic acid, palmitic acid, and linoleic acid were found. Other trace amounts of pentadecanoic acid, tridecanoic acid, margaric acid, eicosadienoic acid, and eicosadienoic acid were identified as well. SeEx and SeEx-C NPs were found to contain both of α- and γ-Linolenic acids.

Alkhoori et al*.*^28^ reported that the concentration and type of amino acids in date seeds depended on their maturation stage. According to Niazi et al*.*^33^, the seed contained high concentration (g/100 g dry seed) of leucine, phenylalanine, and glutamic acid (6.2, 9.8 and16.4 g/100 g). Shina et al*.*^34^ reported that the majority of non-essential amino acids were aspartic acid, alanine and tyrosine (1.6, 1.1 and 1.3 g/100 g, respectively). They added that the majority of essential amino acids were leucine, lysine, and phenylalanine (1.6, 1.2 and 1.1 g/100 g, respectively). Our results were in agreement with the previous researches, where, the ratio of non-essential amino acids to essential amino acids was greater. SeEx and SeEx-C NPs contained all nine necessary amino acids. SeEx and SeEx-C NPs has a high quantity of important amino acids, including lysine, leucine, phenylalanine, and valine. The largest concentration of non-essential amino acids was found in glutamic acid, arginine, and aspartic acid followed by alanine, serine, glycine, and proline.

After determination of the bioactive ingredients in SeEx, it was loaded on C NPs to enhance the delivery of these ingredients to the cells. According to our results, SeEx contained several amino and fatty acids, in addition to, several minerals. Some of these compounds have a high molecular weight that render their entry in the cells leading to the loss of their medical benefits. C NPs can decrease the phytonutrients [contained in SeEx) to the nanoscale, enabling them to enter cells with ease^35^. C is a natural polysaccharide that is prepared from chitin, and it is the most utilised and distributed biomaterial after cellulose^36^. The structure of C is identical to cellulose in structure, however C varies greatly from cellulose in that it also includes acetyl amine, hydroxyl and free amino groups^37^. C is safe, biocompatible and biodegradable; and it has been utilised effectively in the medical industry because it’s not immunogenic and has a mucoadhesive qualities^38^.The C NPs have been loaded with various plant extracts and polyphenols, and have high antioxidant, anticancer, and anti-inflammatory effects when tested in vitro and/or in vivo^39^.

Both C NPs and SeEx-C NPs were observed under SEM and TEM as smooth spherical NPs with size ranges of 63–68 and 81–83 nm, respectively. The Zeta potential for C NPs and SeEx-C NPs, respectively, was 25 and − 55 mV. In a dose-dependent manner, SeEx-C NPs demonstrated a higher antioxidant impact than C NPs and SeEx. SeEx-C NPs shown excellent anti-inflammatory action by inhibiting the hemolysis of red blood cells while protecting Caco-2 cells at high concentrations (up to 250 μg/ml). With increasing the tested dose, SeEx, C NPs, and SeEx-C NPs showed high anti-coagulant activity. SeEx-C NPs had lower PT and PTT values than heparin. From the characters of prepared SeEx-C NPs, it was clear that NPs can be used safely in in vivo testing. The possibility of treating diabetes, in an induced diabetic rat model, by SeEx-C NPs was examined by monitoring FBG and insulin levels. The antioxidant and anti-inflammatory effects of NPs was evaluated in the pancreatic tissue homogenates.

Our results showed that diabetes induction (in untreated diabetic GI) significantly: 1—reduced animals’ body weight, 2—reduced the antioxidant enzymes (SOD, CAT and GSH), 3—elevated MDA level and pro-inflammatory cytokines (IL-1β, TNF-α, IL-6 and IL-4) levels when compared to those of healthy control GI. Moreover, a significant reduction in insulin and C-peptide level; and a significant elevation in FBG and FTA levels was noticed after diabetes induction in GI. SeEx and C NPs (either 10 or 20 mg/kg b.w.) administration lead to an enhancement in animals’ body weight, slight reduction in oxidative stress and inflammation in treated diabetic groups. However, SeEx administration (especially 20 mg/kg b.w.) showed better result than C NPs (either 10 or 20 mg/kg b.w.) administration. Only SeEx-C NPs (either 10 or 20 mg/kg b.w.) have succeeded in decreasing hyperglycemia and increasing insulin level in diabetic treated group. No significant difference in HOMA-IR or QUICKI was observed among the different experimental groups, which can be explained by the type of diabetes induced in this experiment. Single high dose of STZ (65 mg/kg b.w.) induces a type of diabetes that is mimic to type I diabetes^40^, which is characterized by a high FBG and low insulin level due to the destructive effect of STZ on pancreatic β-cells. On the other hand, HOMA-IR and QUICKI are indicators of insulin resistance that are calculated from the fasting insulin and glucose levels; and are suitable markers for type II diabetes more than type I diabetes^21^. Moreover, SeEx-C NPs ameliorated the oxidative stress and inflammation that resulted from STZ administration. This was evident from the significant reduction in MDA and pro-inflammatory cytokines; and the significant elevation in the antioxidant enzymes levels. The histological and immunohistochemical examination of pancreas indicated average-sized normocellular islets of Langerhans with marked insulin immunostain in SeEx-C NPs treated diabetic GVI and GVII. On the other hand, untreated diabetic GI showed markedly damaged hypocellular islets of Langerhans with negative insulin immunostain. SeEx-C NPs administration ameliorated these histological alterations indicating islets cells neogenesis and increased insulin secretion (also indicated from increased C-peptide level).

Our results can be explained by the diabetogenic effects of STZ. The plasma membrane's GLUT 2 glucose transporter allows STZ to be accumulated in pancreatic beta-cells. STZ has a methyl nitrosourea moiety that cause DNA alkylation and fragmentation^41^. The damaged DNA then triggers the DNA repair enzyme poly (ADP-ribose) synthetase leading to ATP and NAD^+^ depletion^42^. Dephosphorylation, which increases the amount of substrates available to xanthine oxidase and causes the production of H_2_O_2_ and –OH radicals, serves as a sign of reduced ATP synthesis^43^. Reactive oxygen species (ROS), reactive nitrogen species (RNS), and the development of inflammatory responses are the mechanisms by which STZ causes beta-cells cytotoxicity^44^. SeEx-C NPs administration significantly ameliorated the diabetogenic effects of STZ. This was evident from the biochemical and histopathological results. Where, SeEx-C NPs (10 and 20 mg/kg b.w.) reduced the oxidative stress and inflammation in rats’ pancreases allowing the islets neogenesis.

The majority of the plants biologically active compounds such as tannins, flavonoids, and terpenoids are soluble in water but have low absorption rates. Because to their big molecular sizes, insufficient absorption, and inability to pass through lipid membranes, these bioactive components' bioavailability and efficiency are diminished. While herbal medicines have good efficacy in in vitro experiments, in vivo examinations are unable to match their effects. According to Ajazuddin^45^, several essential compounds are seldom used due to their ineffectual qualities or problems combining with other ingredients in the formulation. Numerous nanotechnological techniques have removed this barrier by enabling the use of materials with different properties in the same composition and by potentially changing a substance's actions and properties in an organism^46^. In recent years, several studies have concentrated on using nanotechnology in enhancing the delivery of bioactive components in plant extracts. Ghosh et al*.*^47^ reported that nanotechnology elevated the sustained release of these phytochemicals and decreased the possible side effects of using plant extracts. Alharbi et al*.*^48^ used nanotechnology to enhance the topical administration of plants bioactive components. Boosting of the adsorption of green tea and ginseng mixture extract was achieved by using lipid-based structures in the study of Bhattacharya and Ghosh^49^. Also, Gunasekaran et al*.*^50^ showed the enhancement of plants bioavailability through nanophytomedicine development. Therefore, the loading of SeEx on C NPs allowed the delivery of fatty acids (oleic, lauric and myristic acid), amino acids (lysine, leucine, phenylalanine, and valine) and minerals to pancreatic beta-cells in a sustainable manner. SeEx-C NPs have the combined antioxidant and anti-inflammatory effects of SeEx and C NPs, which makes these novel NPs a candidate for diabetes therapy.

## Conclusion

SeEx-C NPs successfully treated STZ-induced diabetes in male Sprague Dawely rats. It increased insulin secretion, allowed pancreatic islets neogenesis and reduced oxidative stress and inflammation. The study has some limitation such as the small number of experimental animals and examination of only two doses (10 and 20 mg/kg b.w.).

## Data Availability

All data of the study will be available from the corresponding author upon reasonable request.

## Abbreviations

DPPH: 1,1-Diphenyl-2-picryl hydrazyl
MTT: 3-(4,5-Dimethylthiazol-2-yl)-2–5-diphenyltetrazolium bromide
Abs: Absorbance
SeEx-C NPs: *Balanites aegyptiaca* seed extract-loaded chitosan nanoparticles
CAT: Catalase
C: Chitosan
C NPs: Chitosan nanoparticles
JNK: C-Jun N-terminal kinase
FBG: Fasting blood glucose
FTA: Fructosamine
GC–MS: Gas chromatography–mass spectrometry
GSH: Glutathione
H&E: Hematoxylin and eosin
ICP: Inductively coupled plasma
IL: Interleukin
MDA: Malondialdehyde
mRNA: Messenger RNA
ANOVA: One way analysis of variance
PTT: Partial thromboplastin time
PT: Prothrombin time
RNS: Reactive nitrogen species
ROS: Reactive oxygen species
SEM: Scanning electron microscope
SD: Standard deviation
STZ: Streptozotocin
SOD: Superoxide dismutase
TEM: Transmission electron microscope
TNF: Tumor necrosis factor
ASK 1: Apoptosis signal-regulating kinase 1
